# Ulcerative colitis, Crohn’s disease, and irritable bowel syndrome have different profiles of extracellular matrix turnover, which also reflects disease activity in Crohn’s disease

**DOI:** 10.1371/journal.pone.0185855

**Published:** 2017-10-13

**Authors:** Joachim Høg Mortensen, Tina Manon-Jensen, Michael Dam Jensen, Per Hägglund, Lone Gabriels Klinge, Jens Kjeldsen, Aleksander Krag, Morten Asser Karsdal, Anne-Christine Bay-Jensen

**Affiliations:** 1 Nordic Bioscience, Biomarkers and Research, Herlev, Denmark; 2 Department of Internal Medicine, Lillebaelt Hospital Vejle, Vejle, Denmark; 3 Department of Biotechnology and Biomedicin, Technical University of Denmark; 4 Odense University Hospital, Department of Gastroenterology, Odense, Denmark; Universitat Hohenheim, GERMANY

## Abstract

**Background:**

Increased protease activity is a key pathological feature of inflammatory bowel disease (IBD). However, the differences in extracellular matrix remodelling (ECM) in Crohn’s disease (CD) and ulcerative colitis (UC) are not well described. An increased understanding of the inflammatory processes may provide optimized disease monitoring and diagnostics. We investigated the tissue remodelling in IBD and IBS patients by using novel blood-based biomarkers reflecting ECM remodelling.

**Methods:**

Five ECM biomarkers (VICM, BGM, EL-NE, C5M, Pro-C5) were measured by competitive ELISAs in serum from 72 CD patients, 60 UC patients, 22 patients with irritable bowel syndrome (IBS), and 24 healthy donors. One-way analysis of variance, Mann-Whitney U-test, logistic regression models, and receiver operator characteristics (ROC) curve analysis was carried out to evaluate the diagnostic accuracy of the biomarkers.

**Results:**

The ECM remodelling was significantly different in UC compared to CD. The best biomarker combination to differentiate UC from CD and colonic CD was BGM and VICM (AUC = 0.98, *P<0*.*001; AUC = 0*.*97*, *P<0*.*001*), and the best biomarker combination to differentiate IBD from IBS patients were BGM, EL-NE, and Pro-C5 (AUC = 0.8, *P<0*.*001*). When correcting for the use of immunosuppressant and elevated CRP levels (CRP>5mg/mL), correlation of Pro-C5 (r = 0.36) with CDAI was slightly improved compared to CRP (r = 0.27) corrected for the use of immunosuppressant. Furthermore, BGM and EL-NE biomarkers were highly associated with colon inflammation in CD patients.

**Conclusion:**

ECM fragments of tissue remodelling in IBD affect UC and CD differently, and may aid in differentiating IBD from IBS (EL-NE, BGM, Pro-C5), and UC from CD patients (BGM, VICM). Formation of type V collagen is related to the level of inflammation in CD and may reflect disease activity in CD.

## Introduction

Inflammatory bowel disease (IBD) is classified as a chronic inflammatory gastro-intestinal (GI) disease [1]. The predominant diseases of IBD are Crohn’s Disease (CD) and ulcerative colitis (UC) [1]. The most common diagnostic tool for IBD is endoscopy, which is invasive, and subjective [2]. Therefore, tissue degradation protein fragments, which are measurable in the serum [3], could be used as objective diagnostic tools in IBD [2]. Serological biomarkers developed for diagnosing IBD and evaluation of complications are currently based on antibodies reacting to yeast glycans and leukocyte antigens e.g. neutrophil granulocytes [4]. Fecal calprotectin[5] is the most commonly used fecal biomarker to differentiate irritable bowel disease (IBS) patients from IBD patients [4]. Biomarkers directly reflecting extracellular matrix (ECM) driven by local tissue inflammation may offer a different class of biomarkers, which may aid in: early diagnosis, identification of progressive disease, and efficacy of medical intervention [6,7].

**Fig 1:**
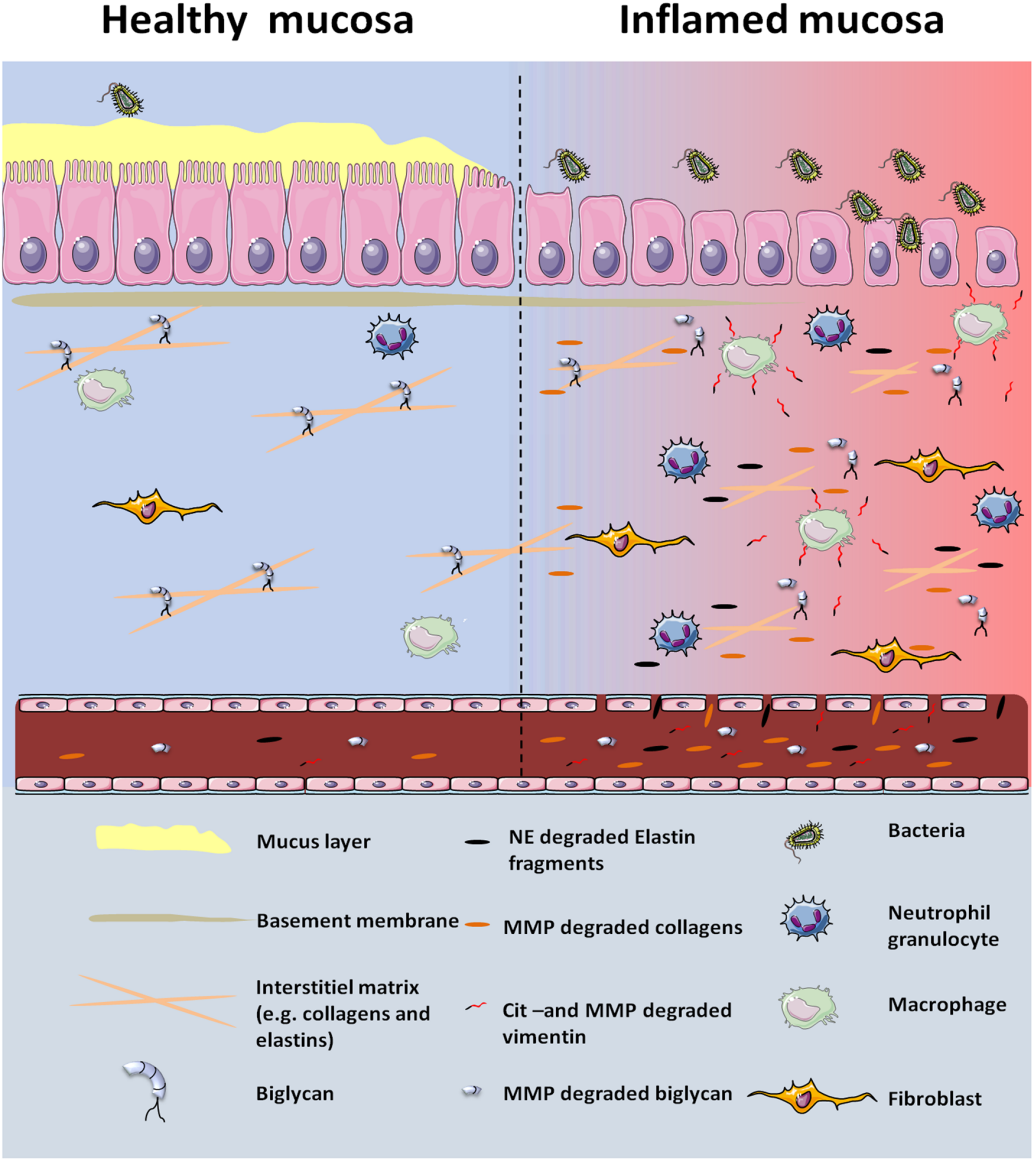
Remodelling of the ECM during the inflammatory process in IBD. The healthy intestinal tissue has intact mucus lining that helps protect the epithelial layer from e.g. bacteria. During the inflammatory state of IBD the mucus layer and the epithelial cell layer is compromised leading to increased bacteria influx. This will, in turn, lead to increased proteinase activity (MMP and NE) and increased infiltration of leukocytes, e.g. macrophages and neutrophil granulocytes. The increased proteinase activity, remodel the ECM that leads to the generation of type V collagen (C5M), biglycan (BGM) and elastin (EL-NE) fragments. Furthermore, local tissue calcium levels also rise as a result of increased cellular turnover. Increased calcium leads to increased citrullination of vimentin that is released from macrophages. Abbreviations: MMP (maxtrix metalloproteinase), NE (neutrolphil elastase), CIT-vimentin (citrullinated vimentin).

The intestinal mucosa consists of an epithelium cell layer overlaying the ECM, composed of basement membrane, interstitial matrix, and fibroblasts [8]. During an inflammatory state, as seen in IBD, infiltration of leukocytes together with fibroblasts alters the ECM turnover and breaks down the matrix into smaller peptide fragments facilitated by increased protease activity e.g. matrix metalloproteinase (MMP) and neutrophil elastase (NE) [9–13]. These protein fragments are released into the circulation where they may be measurable and reflect pathological disease processes (Fig 1) [9–13].

We recently showed that the ECM of the intestinal tissue is affected by the chronic inflammation in IBD, resulting in increased tissue turnover and the release of tissue fragments into the circulation [14]. As a result, the essential relation of the ECM to IBD is not only a consequence of chronic inflammation but may also be part of the pathological process that drives the chronic inflammation in IBD [14]. Therefore other ECM proteins could have relevance in relation to IBD, including biglycan, elastin, and type V collagen which are proteins with essential functions with regards to the ECM [15–18], and expressed in the human colon tract [11,19,20]. Biglycan is a proteoglycan that enables collagen fibrils and elastic fibres assembling and is therefore an essential protein for upholding the ECM (Fig 1) [21,22].

Type V collagen is essential for the organization and formation of fibrils of other collagens in the interstitial matrix; thus type V collagen is partly responsible for the quality and structure of the ECM [18]. Elastin is a major component of elastic fibers which are deposited in the ECM making the tissue elastic [23]. Specific NE-fragments of the elastin are released into the circulation where they can be measured as a biomarker of NE activity and tissue repair [23].

For this study we investigated a panel of novel neo-epitope ECM biomarkers; MMP3 and -9, degraded biglycan (BGM) [24], neutrophil elastase degraded elastin (EL-NE) [23], MMP-9 degraded type V collagen (C5M) [25], and type V pro-collagen (Pro-C5) [26] from different proteinase degradation products and their relation to IBD. The hypothesis was that UC and CD have different patterns of ECM turnover. We sought to investigate three questions: 1) can these biomarkers be used to separate healthy donors from IBD and non-IBD patients? 2) do the biomarkers reflect disease activity, and how do they compare to other biomarkers, e.g. CRP? 3) are the biomarkers related to the location of inflammation in CD patients?

## Materials and methods

### Ethics statement

Ethical approval and consent from patients and healthy donors was obtained prior to enrolling the patients in the original study. Ethical approval for measuring the biomarkers was not acquired for this particular study, since ethical approval for measuring biomarkers in previously collected samples is not required, according to Danish law. Furthermore, only disease relevant information was available for the study at hand. Thus the samples and data were handled anonymously.

### Patient data

The IBD serum samples (collected September 2003 to September 2009), *the Odense University Hospital* (OUH) Cohort, used to investigate the biomarkers in the current study were previously described [14]. Briefly, the cohort included a total of 154 serum samples from CD patients (n = 72, age range [years: 15–75]) (ClinicalTrials.gov Identifier NCT01019460), UC patients (n = 60) (ClinicalTrials.gov Identifier NCT00374725), and patients with IBS (n = 22) (ClinicalTrials.gov Identifier NCT01019460). All patients provided informed consent before participation in the study. Before inclusion of adolescents between 15 and 17 years of age, both the parents and the patient provided informed consent [27]. Disease activity for CD patients was assessed by the Crohn’s disease activity index (CDAI); inactive disease CDAI<150, mild disease activity CDAI = 150–220, moderate/severe disease activity CDAI>220. Disease activity for UC patients was assessed with St. Mark’s score; inactive disease St. Mark’s score<3. We also included 24 serum samples from healthy donors. The serum samples from the OUH cohort had been thawed two times previous to this study. However, all the serum samples were stored at <-20°C. The diagnosis of the IBS patients was based on the ROM-III criteria with standardized clinical assessment, including ileocolonic endoscopy with biopsies, MR-enterography, CT enterography, capsule endoscopy, biomchemistry and faecal assessment for pathogenic gut microflora. IBS patients was neither classified as IBS with diarre or IBS with constipation. The studies was approved by the local ethics committee of Southern Denmark (S-20070072) and the Danish Data Protection Agency (journal number: 2007-41-0675).

#### Healthy donors

Serum from healthy donors was collected in the period of 2012–2013. All healthy donors were negative for GI related diseases and did not show signs of other illness. Standard medical assessment of their health ruled out illnesses. Furthermore, the healthy donors were screened for infectious diseases and found negative for: hepatitis B virus s-antigen, hepatitis C virus, human immunodeficiency virus-1, human immunodeficiency virus-2, human immunodeficiency virus-1 antigen, human immunodeficiency virus-1 nucleic acid testing, and syphilis by FDA-Approved Methods.

### Biomarker assay

The biomarkers included in this study are BGM [24], EL-NE [23], C5M [25], and Pro-C5 [26]. We also included data from the biomarker VICM[28] from a previously published study [14]. The neo-epitope fragments of ECM synthesis and degradation were assessed by solid phase competitive enzyme-linked immunosorbent assays (ELISAs) as previously described [14]. These assays were based on monoclonal neo-epitope antibodies which recognize a specific amino acid sequence that is generated by cleavage of a protein by a specific protease. Futhermore, these biomarker assays recognizes only the exact cleavage site of the protein fragment, however the neo-epitope fragment can be different sizes. Thus these assays can only be used to quantify the presence of the neo-epitope not the size of the neo-epitope. The current manuscript is not a dual publication since it has included new biomarkers not yet investigated in inflammatory bowel disease.

Briefly, streptavidin pre-coated 96-well plates (Roche Diagnostic’s cat. No. 11940279, Hvidovre, Denmark) were coated with a biotinylated antigen for 30 minutes at room temperature. After adding the calibrator, kit controls and serum samples to the coated wells, horseradish peroxidase-conjugated monoclonal antibodies were added to the wells and incubated for 1–3 hours at 4°C/20°C or 20 hours at 4°C depending on the specific assay. Tetramethylbenzidine (TMB, Kem-En-Tec cat. No. 438OH, Taastrup, Denmark) was added and incubated for 15 minutes at room temperature and agitated at 300 rpm. The TMB reaction was stopped by addition of a stopping buffer (1% H_2_SO_4_). Wells were thoroughly washed after each incubation step with buffer (25 mM TRIZMA, 50 mM NaCl, 0.036% Bronidox L5, 0.1% Tween 20) using a standardized ELISA plate washing machine (BioTek^®^ Instruments, Microplate washer, ELx405 Select CW, Winooski, USA). The optical densities were read at 450 nm and 650 nm as reference using ELISA reader (VersaMAX; Molecular Devices, Wokingham Berkshire, UK). A standard curve was plotted using a 4-parametric mathematical fit model.

#### Statistical analyses

The biomarkers were log-transformed to achieve normal distribution. Therefore, one-way ANOVA with Bonferroni correction and student t-test was applied. The biomarker levels were presented as means with standard error of the mean. To evaluate the discriminative power of the biomarkers, receiver operator characteristic-curves (ROC-curve) was calculated and backwards logistic regression analyses were carried out to calculate the best diagnostic value by combining all the markers and correcting for confounding factors (age, BMI, gender, smoking, use of immunosuppressant drugs, disease activity, and Montreal classification). The diagnostic accuracy was calculated by the following equation: Diagnostic accuracy = ((True Negatives + True Positives)/(All Patients)). A *P*-value ≤0.05 was considered statistically significant. Biomarkers were divided into 25% quartiles (Q1-Q4: where Q1 represents CD patients with the 25% lowest biomarker levels of the CD cohort, and Q4 represent CD patients with the highest 25% highest biomarker levels of the CD cohort), the sum of quartiles (the sum of quartiles was calculated by adding the quartiles of two or more biomarkers thus leading to a sum of quartiles from 2–8) and cumulative quartiles (the sum of quartiles was divided into the four quartiles. Q1 = sum of quartiles of 2, Q2 = sum of quartiles of 3–4, Q3 = sum of quartiles to 5–6, Q4 = sum of quartiles of 7–8) was calculated to assess their association with disease location in CD patients. Fisher’s exact test and odds ratios (OR) were calculated to investigate the relationship between the number of biomarkers elevated in CD patients and disease location. Mean serum levels of the biomarkers with standard diviation (SD) can be found in Tables A, B, C, and D in S1 File. For statistical calculation and figure editing MedCalc v.14.8.1., and Graphpad Prism v.6 were applied.

## Results

### Cohort description

This study included 72 CD patients, 60 UC patients, 22 IBS patients, and 24 healthy donors. The groups were of similar age (mean age 41.3 years), but gender was not matched (Table 1). About 71% of IBD patients had clinically active disease.

**Table 1:** Patient demographics.

	Odense University Hospital cohort			
	CD (n = 72)	UC (n = 60)	IBS (n = 22)	Healthy donors (n = 24)
**Gender**				
• **Male, (%)**	27 (37)	29 (48)	2 (9)	21 (88)
• **Female, (%)**	45 (63)	31 (52)	20 (91)	3 (12)
**Age mean years; (range)**	35.8 (15–76)	37.3 (21–70)	34.4 (16–75)	41.3 (19–60]
**BMI mean kg/m**^**2**^**; (range)**	25.8 (16.2–45.1)	26 (18.6–36.4)	26.3 (18.1–43.3)	N/A
**Smoking**				
• **Yes, (%)**	40 (56)	35 (58)	9 (41)	N/A
• **No, (%)**	32 (44)	25 (42)	13 (59)	N/A
Use of immunosuppressants*				
• **Yes, (%)**	30 (42)	32 (53)	0 (0)	N/A
• **No, (%)**	42 (58)	28 (47)	22 (100)	N/A
**Bowel resection prior to inclusion**	20	0	0	0
**Disease activity**	CDAI score	St. marks score		
• **Active disease, (%)**	37 (51)	54 (90)	N/A	N/A
• **Remission, (%)**	30 (42)	6 (10)		

Abbreviations: CD; Crohn’s disease, UC; ulcerative colitis, IBS; irritable bowel disease, N/A; not available.

The total overview of patients demographics can be reviewed in a previously published article[14].

*use of immunosupressants/glucocorticoids.

### Level of the BGM, EL-NE, C5M, and Pro-C5 biomarkers in IBD

**Fig 2:**
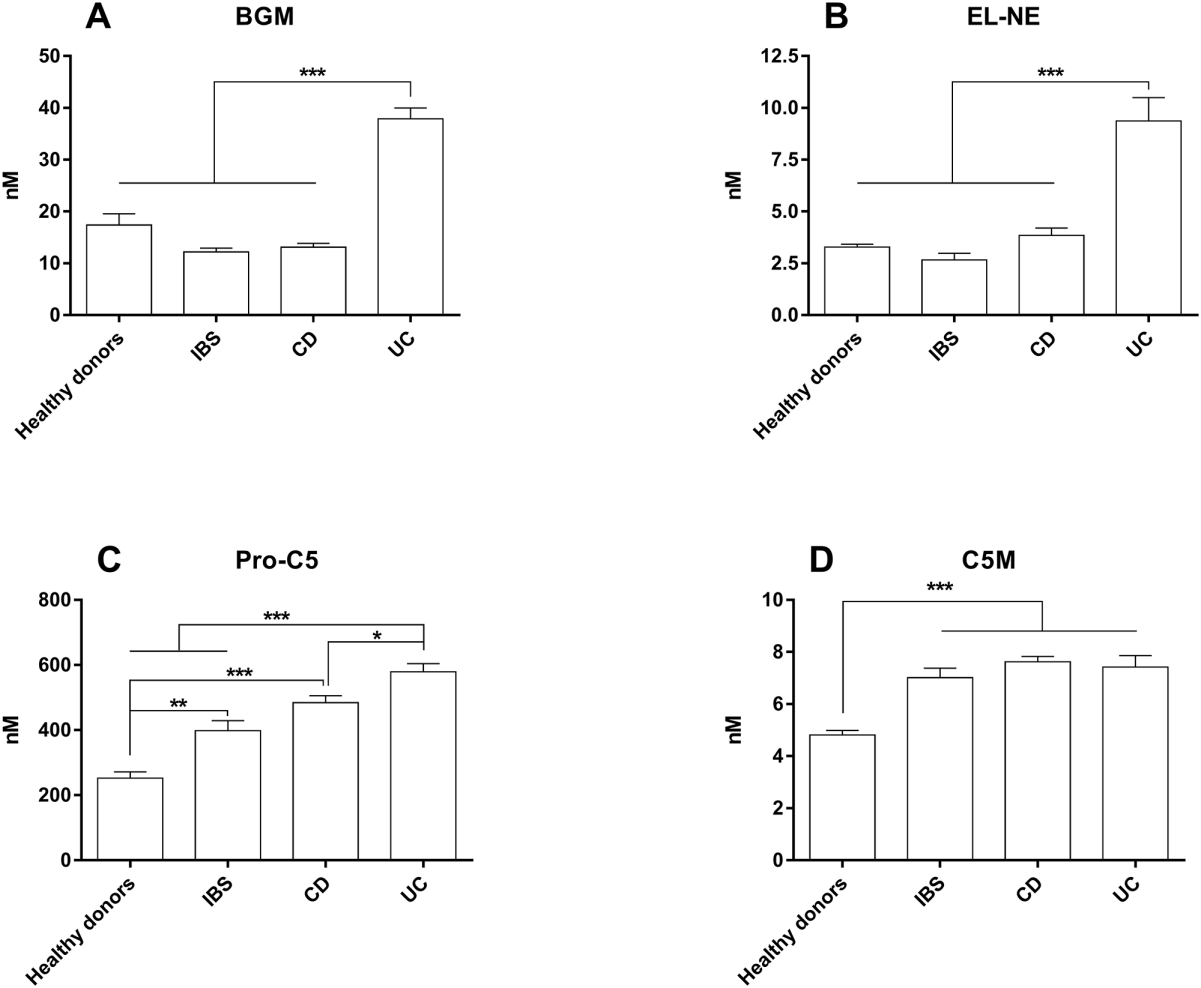
*S*erum levels of the ECM serum biomarkers BGM, EL-NE, Pro-C5, and C5M in CD, UC, IBS and healthy controls. **A**) BGM serum level, **B**) EL-NE serum levels, **C**) Pro-C5 serum levels, and **D**) C5M serum level. The error bars represent standard error of the mean (SEM). The asterisks (*) represent *P-values*, *P<0.*05*, **P<0.*01*, ***P<0.*001*.

The MMP degraded biglycan biomarker BGM and the NE degraded elastin biomarker EL-NE were significantly elevated in UC compared to CD (*P<0*.*001*) and IBS (*P<0*.*001*) patients (Fig 2A and 2B). In addition, BGM was significantly elevated in UC patients compared to healthy donors (p = 0.005) (Fig 2A). Pro-C5 was significantly higher in both UC, CD, and IBS patients than in healthy donors (CD and UC: *P<0*.*001*, IBS: *P<0*.*01*), and UC patients had significantly elevated levels compared to IBS patients (*P = 0*.*019*) (Fig 2C). C5M was significantly elevated in IBD and IBS patients compared to healthy donors (*P<0*.*001*) (Fig 2D). The CRP levels were not different between UC, CD or IBS patients (S1 Fig).

### Diagnostic accuracy for discriminating disease groups

The diagnostic accuracies of the investigated biomarkers were evaluated in terms of their discriminative power to differentiate CD from UC and IBD from IBS patients.

### UC vs. CD

BGM and EL-NE were the best biomarkers for differentiating UC from CD patients with diagnostic accuracies (DA) of 90% and 75%, respectively (Table 2). Logistic regression was used to investigate the best combination biomarkers, which was BGM and VICM with a diagnostic accuracy of 94% to differentiate UC from CD. The diagnostic accuracy of BGM and VICM in combination was also calculated in UC vs. colonic CD, showing a diagnostic accuracy of (AUC = 0.97, DA = 94%) (Table 2).

**Table 2:** Shows the AUC, sensitivity, and specificity, of each ROC-analysis and percentage of cases correctly identified, and combination of biomarkers.

	AUC (CI)	P values	Sens. %	Spec. %	PPV. %	NPV. %	DA %
**Biomarkers: UC (n = 60) vs. CD (n = 72)**							
BGM	0.95 (0.90–0.98)	*<0*.*001*	93	87	86	94	90
EL-NE	0.85 (0.78–0.91)	*<0*.*001*	78	78	74	81	75
C5M	0.61 (0.52–0.69)	*<0*.*05*	48	76	63	64	55
Pro-C5	0.65 (0.56–0.73)	*<0*.*01*	85	43	55	78	60
VICM, see Mortensen et al. [14]							
CRP	0.56 (0.47–0.65)	*NS*	100	39	56	100	54
BGM + VICM*	0.98 (0.93–1.00)	*<0*.*001*	94	96	94	96	94
**Biomarkers: UC (n = 60) vs. Colonic CD (n = 48)**							
BGM	0.94 (0.88–0.98)	*<0*.*001*	83	95	93	88	88
EL-NE	0.87 (0.79–0.93)	*<0*.*001*	60	93	88	74	87
C5M	0.62 (0.53–0.72)	*NS*	79	52	57	76	52
Pro-C5	0.69 (0.59–0.78)	*<0*.*01*	79	48	55	74	69
VICM, see Mortensen et al. [14]							
CRP	0.52 (0.39–0.69)	*NS*	45	54	43	60	53
BGM+VICM*	0.97 (0.92–1.0)	*<0*.*001*	96	91	90	96	94
**Biomarkers: IBD (n = 132) vs. IBS (n = 22)**							
BGM	0.70 (0.62–0.78)	*<0*.*001*	60	91	98	28	85†
EL-NE	0.77 (0.69–0.83)	*<0*.*001*	78	65	93	34	85†
C5M	0.56 (0.48–0.64)	*NS*	62	57	89	21	85†
Pro-C5	0.71 (0.59–0.83)	*<0*.*001*	78	61	92	33	85†
VICM	0.59 (0.51–0.66)	*NS*	83	41	89	29	85†
CRP	0.51 (0.43–0.59)	*NS*	51	58	88	17	85†
EL-NE+BGM+Pro-C5*	0.83 (0.76–0.88)	*<0*.*001*	68	91	98	32	85†
**Biomarkers: IBD (n = 132) vs. healthy donors (n = 24)**							
BGM	0.61 (0.53–0.69)	*<0*.*01*	46	79	92	21	85†
EL-NE	0.65 (0.57–0.72)	*<0*.*01*	52	96	99	27	85†
C5M	0.91 (0.85–0.95)	*<0*.*001*	84	92	98	51	85†
Pro-C5	0.93 (0.88–0.96)	*<0*.*001*	86	88	97	54	85†
C5M+Pro-C5+EL-NE*	0.98 (0.95–0.99)	*<0*.*001*	86	88	97	54	85†
**Biomarkers: IBS (n = 22) vs. healthy donors (n = 24)**							
BGM	0.64 (0.49–0.78)	*<0*.*05*	91	58	67	88	64
EL-NE	0.75 (0.60–0.86)	*NS*	68	96	83	75	76
C5M	0.92 (0.80–0.98)	*<0*.*001*	96	83	84	96	92
Pro-C5	0.83 (0.69–0.92)	*<0*.*001*	96	63	70	94	83
C5M+Pro-C5+EL-NE*	0.96 (0.86–1.0)	*<0*.*001*	96	88	88	96	98

Abbreviations: AUC: Area under the curve, CI: Confidence interval, Sens: Sensitivity, Spec: Specificity, PPV: Positive predictive value, NPV: Negative predictive value, DA: Diagnostic accuracy

* Best logistic regression model with combination of biomarkers.

^†^sample size is too low for the control groups (IBS and healthy donors) resulting in overestimation of the diagnostic value.

### IBD vs. IBS

None of the biomarkers alone could achieve an AUC of more than 0.77 for separating IBD from IBS. However, the combination of BGM, EL-NE, and Pro-C5 improved the AUC to 0.83 for differentiating IBD from IBS patients. Due to low sample size of the IBS patient group the DA may be overestimated (Table 2).

#### IBD and IBS vs. Healthy donors

All biomarkers demonstrated statistical significant clinical discrepancy to differentiate IBD from healthy donors. The combination between Pro-C5, C5M, and EL-NE, was the best to differentiate IBD from healthy donors (AUC = 0.98). Due to low sample size of the IBS patient group the DA may be overestimated (Table 2). For IBS patients, C5M and Pro-C5 demonstrated statistical significant clinical discrepancy to differentiate IBS from healthy donors. However, the best combination of biomarkers to differentiate between IBS patients and healthy donors was C5M, Pro-C5 and El-NE (Table 2).

All regression models were adjusted for confounders; no confounding variables affected the logistic regression model. ROC-curves for every biomarker and combination of biomarkers can be seen in the supplementary data (S1 Fig and Fig 2).

### Formation of type V collagen and CRP are descriptive of disease activity

#### Pro-C5 and CRP levels in mild-moderate and severe disease activity

**Fig 3:**
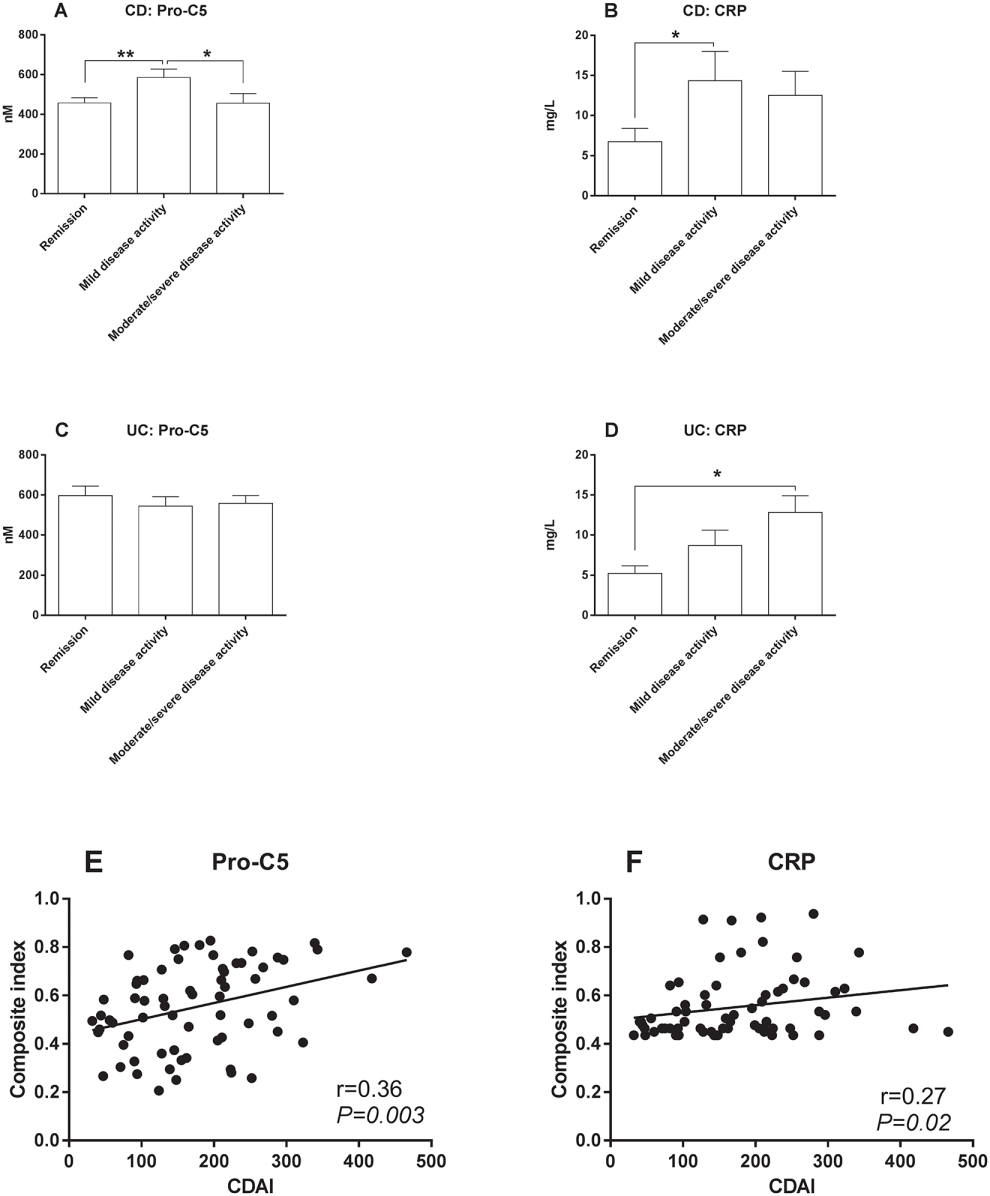
*S*erum levels of the biomarkers Pro-C5 and CRP in CD UC patients and correlation of Pro-C5 and CRP in relation to disease activity. For CD patients the disease activity was assessed by the Crohn’s disease disease acitivity index (CDAI). Remission < CDAI 150, mild = CDAI 150–220, moderate/severe > CDAI 220. Disease activity for UC patients was based on the st. Marks score. Remission < score 3, Mild = score 3–4, Moderate/severe > score 4. **A**) Pro-C5 serum levels in CD patients, **B**) CRP levels in CD patients, **C**) Pro-C5 serum levels in UC patients, **D**) CRP serum levels in UC patients, **E**) correlation of Pro-C5 with CDAI as compostite index of the predicted values from the logistic regression model when correcting for CRP levels >5 and use of immunosuppressant drugs, and **F**) correlation of CRP with CDAI as compostite index of the predicted values from the logistic regression model when correcting for use of immunosuppressant drugs. The error bars represent standard error of the mean (SEM). The asterisks (*) represent *P-values*, *P<0.*05*, **P<0.*01*.

Only Pro-C5 and CRP showed association with disease activity in CD (Fig 3). CD patients with mild disease activity had significantly elevated levels of Pro-C5 compared to CD patients in remission (P<0.01) and CD patients with moderate/severe disease activity (P<0.05) (Fig 3). No clinical relevance was observed in UC patients with regards to Pro-C5 and disease activity (Fig 3D). In addition, CRP levels were significantly elevated in patients with active disease for both UC and CD

#### Correlation of Pro-C5 and CRP with CDAI in CD

In an attempt to utilize the full potential of Pro-C5 as a biomarker of disease activity in CD, a composite index including Pro-C5 and potential confounding variables was tested. The use of immunosuppressants and elevated CRP levels (CRP>5mg/L) were the only confounders that significantly affected Pro-C5 (Fig 3E) and use of immunosuppressants affected CRP (Fig 3F). A better correlation between CDAI and Pro-C5 was seen when adjusting for CRP>5mg/L and use of immunosuppressant (Fig 3E).

### Association between the biomarkers and location of disease in Crohn’s disease

**Fig 4:**
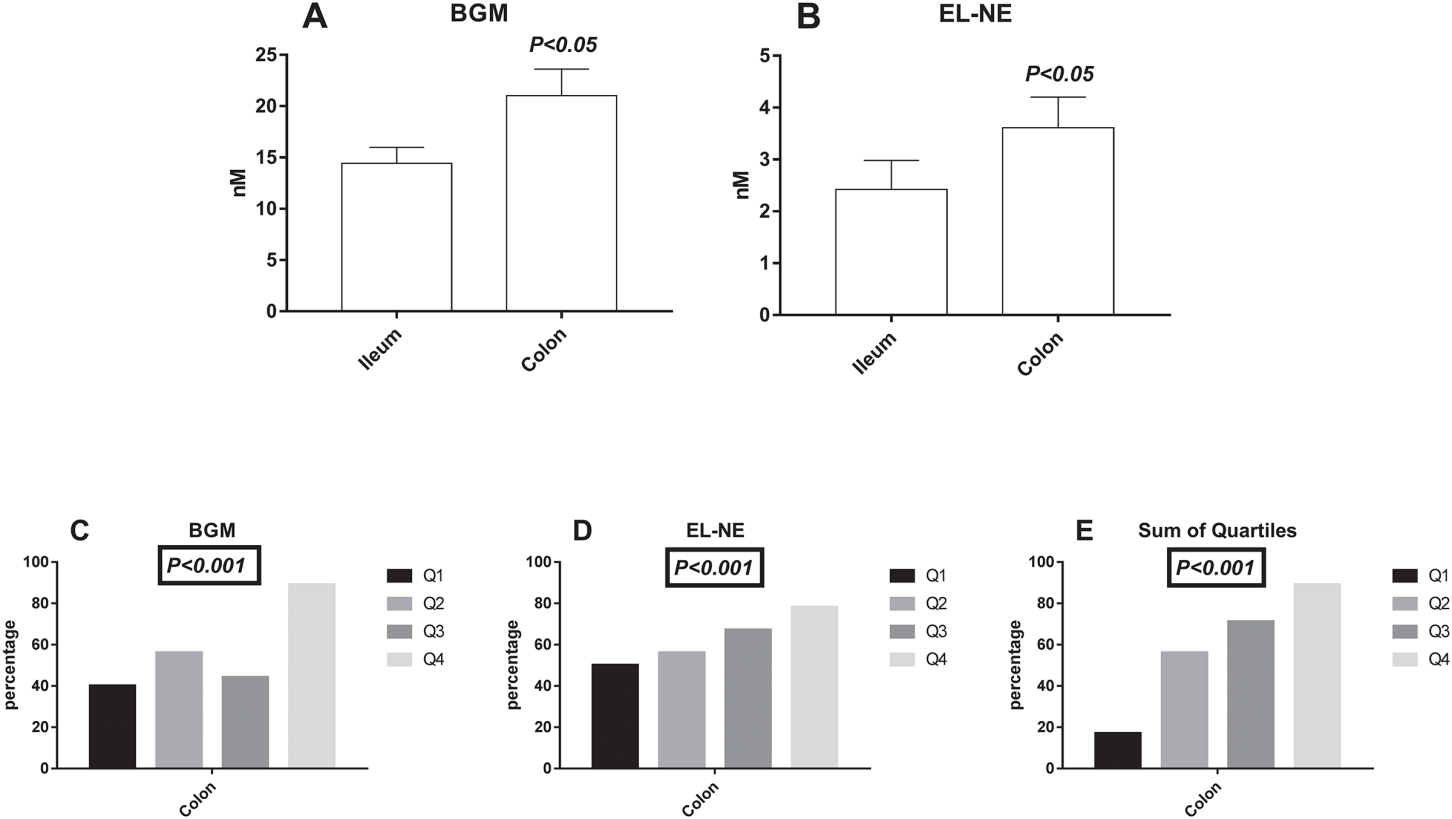
*S*erum levels of the ECM serum biomarkers BGM and EL-NE in relation to location in Crohn’s disease and the percentage of colon involvement as a function of the biomarker levels. The biomarkers were divided into 25% quartiles (Q1, Q2, Q3, Q4), and consisted of 10, 9, 9, and 9 patients respectively. The cumulative sum of the both biomarkers was calculated in divided into quartiles of sum (Q1, Q2, Q3, Q4), and consisted of 5, 9, 13, and 9 patients respectively. A) BGM serum level in patients with colon and ileum involvement, B) EL-NE serum levels with colon and ileum involvement, C) percentage of colon involvement for BGM Q1, Q2, Q3, and Q4, D) percentage of colon involvement for EL-NE Q1, Q2, Q3, and Q4, E) percentage of colon involvement for the sum of quartiles Q1, Q2, Q3, and Q4. The error bars represent standard error of the mean (SEM). Student t-test was applied for figures A and B, the chi-squared test was applied for the figures C, D, and E.

CD patients with ileum involvement (L1) had significantly lower levels (*P<0*.*05*) of BGM (Fig 4A), and EL-NE (Fig 4B) compared to CD patients with colon involvement (L2, L3). To investigate the biomarkers’ relation to colon involvement, BGM and EL-NE was divided into 25% quartiles (Q1-Q4), and the cumulative quartiles was also calculated for these biomarkers. The quartiles for the biomarkers and the cumulative quartile demonstrated that Q2-Q4 had increased association of colon involvement compared to Q1 (*P<0*.*001*) (Fig 4C, 4D and 4E). The probability of colon involvement was 12 times higher in the Q4 BGM quartile compared to Q1 (*P<0*.*027*)(Table 3). The cumulative quartiles also demonstrated that the probability of having colon involvement was increased in Q4 (OR: 40, *P<0*.*006*), and Q3 (OR: 12.5, *P = 0*.*024*) compared to Q1 (Table 3).

**Table 3:** Shows the probability of having colon involvement in Crohn’s disease patients with active disease, by dividing the biomarkers BGM and EL-NE into 25% quartiles and also the sum of quartiles.

	Q1 vs. Q2		Q1 vs. Q3		Q1 vs. Q4	
	OR (CI)	P-value	OR (CI)	P-value	OR (CI)	P-value
• **BGM**	1.9 (0.30–11.6)	*0*.*754*	1.2 (0.19–7.44)	*0*.*787*	**12 (1.05–137)**	***0*.*027***
• **EL-NE**	1.3 (0.21–7.62)	*0*.*83*	2.0 (0.31–12.8)	*0*.*46*	3.5 (0.47–25.9)	*0*.*210*
• Sum of quartiles*	6.25 (0-50-77.5)	*0*.*13*	**12.5 (1.09–143)**	***0*.*024***	**40 (2.01–794)**	***<0*.*006***

Abbreviations: OR: Odds ratio, CI: confidence interval, BM: Biomarker. Significant odds ratios are highlighted in bold.

*quartiles of the cumulative quartiles from BGM and EL-NE

## Discussion

The current study presents the result of five blood-based biomarkers directly reflecting ECM turnover (BGM, C5M, Pro-C5), and indirectly neutrophil (EL-NE) and macrophage (VICM) activity capable of differentiating UC from CD, and IBD from IBS patient, clinically active and inactive CD, and location of disease in CD.

The biomarkers BGM, EL-NE, and Pro-C5 were associated with UC, and VICM [14] was associated with CD. By combining VICM [14], and BGM high clinical accuracy to differentiate UC from CD was achieved with an AUC of almost 1. These results suggest a high clinical relevance for differentiating CD and UC, and may aid clinicians in diagnosing IBD. Additionally, the serum BGM levels for CD reported in this study are in concordance with the recent publication by Goffin et al. [29], indicating that the biomarker BGM is indeed more associated with UC than CD. Furthermore, the type V collagen formation biomarker, Pro-C5, was significantly elevated in IBD patients compared to IBS patients, and when combined with BGM and EL-NE, the biomarkers were able to differentiate IBD patients from IBS patients, which imply that these biomarkers can be used to exclude IBS patients for IBD diagnostics. Interestingly the degradation of type V collagen, C5M, and formation of type V collagen, Pro-C5, was found to be elevated in IBD patients as well as in IBS patients compared to healthy donors. It was expected that the C5M and Pro-C5 biomarkers were increased in IBD compared to IBS patients, because of the chronic inflammatory state in IBD. The best combination of biomarkers to differentiate between IBS patients and healthy donors was C5M, Pro-C5, and EL-NE with high diagnostic accuracy. These data may indicate that IBS patients, to some extent, could have micro-inflammation that in turn could lead to increased ECM remodelling in patients with IBS.

A biomarker diagnostic platform was recently developed by Prometheus laboratories, combining 17 serological, genetic, and inflammatory (SGI) biomarkers which may provide clinicians with a tool to facilitate the diagnosis and stratification of IBD patients into treatment and risk groups for a more streamlined patient care [30]. However, the entire diagnostic accuracy of the SGI diagnostic platform may account for only 3 of the serological biomarkers, namely the pANCA, ASCA-IgA, and ASCA-IgG [30]. Our results indicate that by combining up to 3 ECM neo-epitope biomarkers, high diagnostic accuracy can also be achieved.

In addition, biglycan fragments may act as a chemokine [31], and is also a ligand of toll-like receptors-2/4 [31,32] in particular. Cario et al. [33] demonstrated that the expression of TLR-4 differed between CD and UC, and that CD patients had the most intense staining at the apical pole of the epithelial cells, while UC patients had highly intense TLR-4 expression at the basolateral pole, however apical staining was also observed in UC patients. Thus, the observation that UC patients have elevated levels of degraded biglycan (BGM), irrespective of disease activity, may support the fact that fragments of biglycan are a ligand of TLR-4 and contributes to a continuous, chronic inflammatory state in UC patients [34].

Involvement of neutrophil granulocytes in IBD has previously been described to be more pronounced in patients with UC compared to CD [35]. Evidence suggests that both the recruitment of neutrophil granulocytes and migration are decreased in CD patients [35]. Thus biomarkers that are linked to neutrophil granulocyte activity are potential biomarkers for UC. Bennike et al., demonstrated that NE and MMP-9 were highly elevated in the mucosa of UC patients compared to healthy controls. However, elastin expression in the intestinal mucosa was lower in the UC patients compared to the healthy controls, indicating increased degradation of elastin [11], which is in line with the EL-NE data presented in this study.

Pro-C5 was significantly decreased in CD patients in remission compared to CD patients with mild disease activity. In contrast, the CRP levels were only significantly different in CD patients in remission compared to CD patients with mild disease activity. We present for the first time that ECM biomarker of type V collagen formation (Pro-C5), may improve the monitoring of disease activity with a synergistic effect of CRP. In addition, Haaften et al. showed that Pro-C5 proved significantly more associated with CD patients with fistulising disease behaviour than with other behavioural phenotypes of CD [36].

The inflammatory nature in IBD is different in UC and CD. In UC the inflammation affects superficial layers of mucosa, from the rectum to proximal parts of the colon—in some patients involving the entire colon (pancolitis) [1]. In CD, on the other hand, skip lesions often occur and the inflammation can affect smaller parts of the entire GI tract simultaneously [37]. Therefore, the differences in the observed levels of serum BGM and EL-NE levels could be explained by the difference in the GI inflamed surface area and the composition of the ECM in the colon and ilieum. For CD patients with active disease higher serum levels of the biomarkers BGM and EL-NE were associated with colon involvement. As both the biomarkers BGM and EL-NE was highly increased in UC compared to CD patients, these data further support the relation of the BGM and EL-NE to UC since UC exclusively affects the colonic tissue. Furthermore, the diagnostic accuracy of the combination of BGM and VICM to differentiate UC from colonic CD (94% DA) was identical to the combination of BGM and VICM to differentiate UC from CD with both ileal and colonic involvement (94% DA).

There are several limitations to the current study. The study is lacking a validation cohort. However, the serum BGM levels in CD patients was confirmed by a recent study by Goffin et al. [29]. However, these biomarkers have been thoroughly validated and normal serum levels have previously been reported, and a clinical relevant IBS cohort was included as well as healthy controls. The study population is very heterogeneous and was not designed for the current study. The CRP levels were similar in the IBD patients as well as the IBS patients. As IBS patients do not have increased inflammation, the IBS patients included in this study may not be the optimal representable IBS patient group. Therefore, a more clear and defined patient cohort of IBD and IBS is desirable to fully investigate the tissue turnover in IBD and IBS patients.

## Conclusion

Collectively, the current study shows very promising results in regards to differentiating CD from UC, and IBD from IBS patients. By combining the biomarkers BGM and VICM high, diagnostic accuracy is achieved to differentiate UC from CD, and the combination of Pro-C5 together with BGM and EL-NE biomarkers demonstrated high diagnostic accuracy to differentiate IBD from IBS patients. In addition, the formation biomarker of type V collagen, Pro-C5 has a synergistic effect with CRP in patients with CD and may aid and improve the monitoring of disease activity in CD. The biomarkers BGM, EL-NE, and VICM serum levels differ in IBD patients and may be related to the aetiology of IBD.

## Supporting information

S1 Data The data for each assay measured in serum from the included patients can be reviewed in S1 Data.xlsx’ file. Data for the VICM data can be reviewed in the original article by Mortensen et al. [14].
(XLSX)

S1 Fig *S*erum levels of C-reactive protein (CRP) in CD, UC, and IBS patients. The error bars represent standard error of the mean (SEM).
(TIF)

S2 Fig Receiver operator characteristic curve (ROC-curve) of each ECM serum biomarker, BGM (blue curve), EL-NE (red curve), C5M (orange curve), and Pro-C5 (green curve). A) ROC-curve of the biomarkers in UC patients (n = 60) vs. CD patients (n = 72), B) ROC-Curve of the biomarkers in IBD patients (n = 132) vs. IBS patients (n = 22). C) ROC-Curve of the biomarkers in IBD patients (n = 132) vs. healthy donors (n = 24).
(PNG)

S3 Fig Receiver operator characteristic curve (ROC-curve) of the best combination of biomarkers in a logistic regression model, plotted as predicted values from the logistic regression model, do differentiate UC (n = 60) patients from CD patients (n = 72), and IBD patients from IBS patients. **A**) Combination of the biomarkers (BGM and VICM†) to differentiate CD patients from UC patients patients. **B**) Combination of biomarkers (EL-NE, Pro-C5, and BGM) to differentiate IBD patients (n = 132) vs. IBS patients (n = 22). **C**) Combination of biomarkers (C5M and BGM) to differentiate IBD patients (n = 132) vs. healthy donors (n = 24).
(PNG)

S1 File Tables A, B, C, and D. Mean and standard deviation of all the biomarkers.
(DOCX)

## Abbreviations

ASCA: Anti-Saccharomyces cerevisiae
AUC: Area under the curve
BGM: Maxtrix metalloproteinase degraded biglycan
BM: Biomarker
BMI: Body mass index
C5M: Maxtrix metalloproteinase degraded type V collagen
CD: Crohn’s disease
CDAI: Crohns disease activity index
CI: confidence interval
CRP: C-reactive protein
DA: diagnostic accuracy
ECM: Extracellular matrix
ELISA: Enzyme-linked immunosorbent assays
EL-NE: Neutrophil elastase degraded elastin
IBD: Inflammatory bowel disease
IBS: Irritable bowel syndrome
MMP: Maxtrix metalloproteinase
N/A: Not available
NE: Neutrophil elastase
NPV: Negative predictive value
OR: Odds ratio
OUH cohort: Odense University Hospital cohort
pANCA: perinuclear anti-neutrophile cytoplasmic antigen
PPV: Positive predictive value
Pro-C5: formation of type V collagen
Q1: Quartile 1
Q2: Quartile 2
Q3: Quartile 3
Q4: Quartile 4
ROC-curve: receiver operator characteristics-curve
Sens: Sensitivity
Spec: Specificity
TMB: Tetramethylbenzidine
UC: Ulcerative colitis
VICM: citrullinated and Matrix metalloprotease degraded vimentin
