# Supplementary figures and images for: Ulcerative colitis, Crohn’s disease, and irritable bowel syndrome have different profiles of extracellular matrix turnover, which also reflects disease activity in Crohn’s disease
Source: PLoS One. 2017 Oct 13;12(10):e0185855. doi: 10.1371/journal.pone.0185855 (PMC5640222; doi:10.1371/journal.pone.0185855)

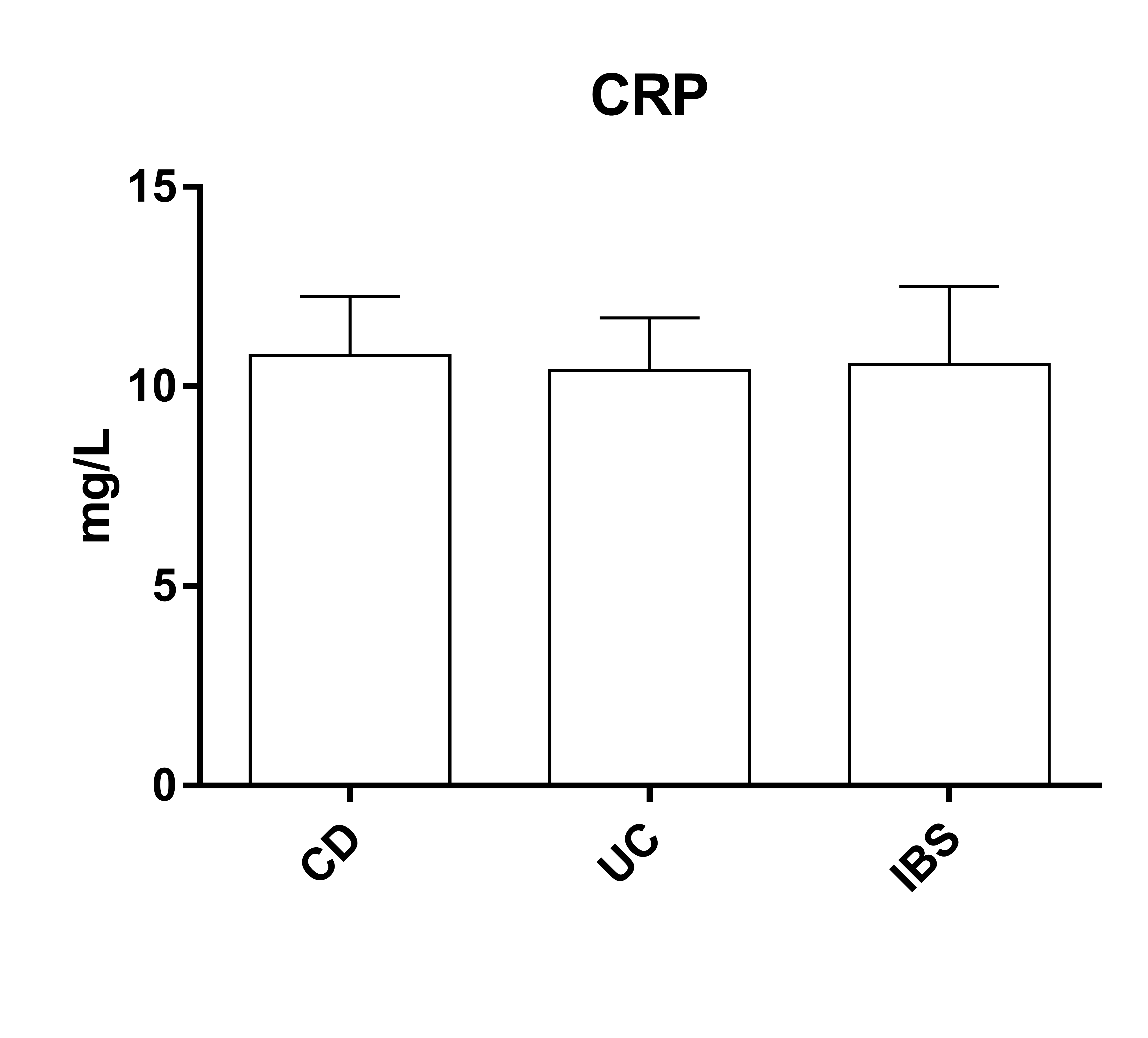

Supplement: S1 Fig — The error bars represent standard error of the mean (SEM). (TIF) [file pone.0185855.s002.tif]

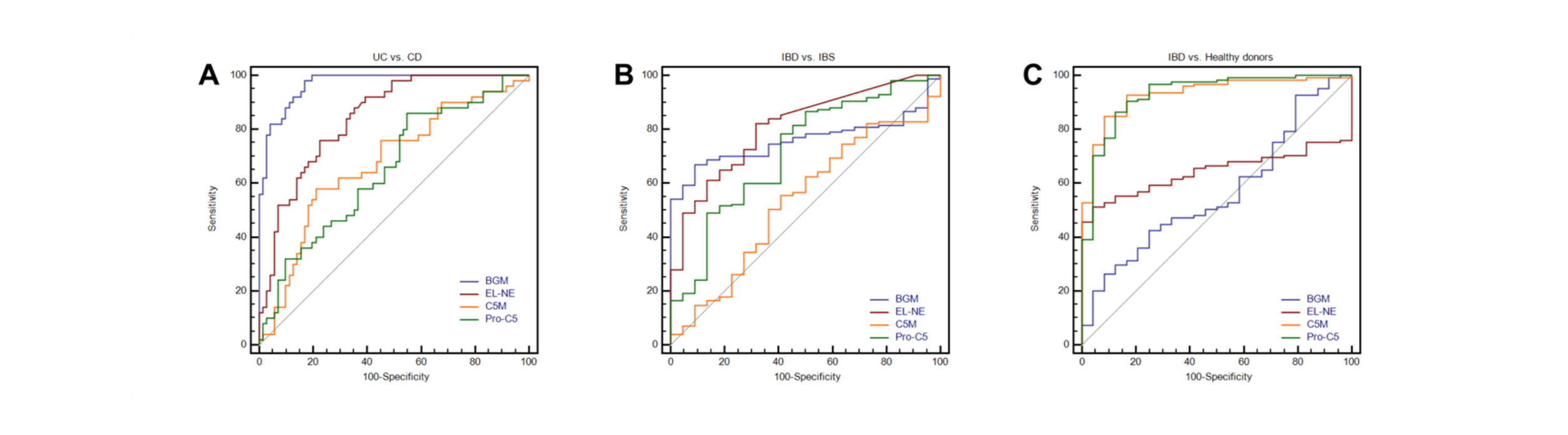

Supplement: S2 Fig — A) ROC-curve of the biomarkers in UC patients (n = 60) vs. CD patients (n = 72), B) ROC-Curve of the biomarkers in IBD patients (n = 132) vs. IBS patients (n = 22). C) ROC-Curve of the biomarkers in IBD patients (n = 132) vs. healthy donors (n = 24). (PNG) [file pone.0185855.s003.png]

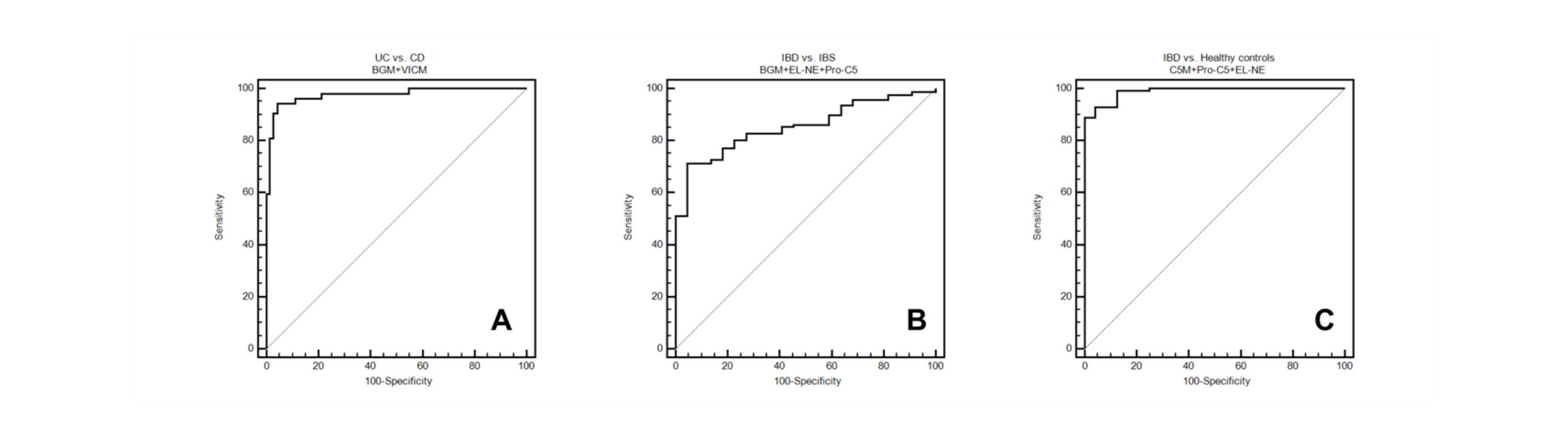

Supplement: S3 Fig — A) Combination of the biomarkers (BGM and VICM†) to differentiate CD patients from UC patients patients. B) Combination of biomarkers (EL-NE, Pro-C5, and BGM) to differentiate IBD patients (n = 132) vs. IBS patients (n = 22). C) Combination of biomarkers (C5M and BGM) to differentiate IBD patients (n = 132) vs. healthy donors (n = 24). (PNG) [file pone.0185855.s004.png]
