# Supplementary material for: Ulcerative colitis, Crohn’s disease, and irritable bowel syndrome have different profiles of extracellular matrix turnover, which also reflects disease activity in Crohn’s disease
Source: PLoS One. 2017 Oct 13;12(10):e0185855. doi: 10.1371/journal.pone.0185855 (PMC5640222; doi:10.1371/journal.pone.0185855)
Supplement: S1 File — Mean and standard deviation of all the biomarkers. (DOCX) [file pone.0185855.s005.docx]

| **Supplementary table A: The biomarker BGM, mean and standard deviaton** | | | | |
| --- | --- | --- | --- | --- |
|  | **Healthy donors** | **irritable bowel syndrome** | **Crohn's disease** | **Ulcerative colitis** |
| **Mean** | 22,63 | 15,85 | 17,04 | 49,28 |
| **Standard deviation** | 13,72 | 4,635 | 8,288 | 20,96 |
|  |  |  |  |  |
|  |  |  |  |  |
|  |  |  |  |  |
|  |  |  |  |  |
| **Supplementary table B: The biomarker EL-NE, mean and standard deviaton** | | | | |
|  | **Healthy donors** | **irritable bowel syndrome** | **Crohn's disease** | **Ulcerative colitis** |
| **Mean** | 2,726 | 2,208 | 3,192 | 7,776 |
| **Standard deviation** | 0,5605 | 1,255 | 2,525 | 7,083 |
|  |  |  |  |  |
|  |  |  |  |  |
|  |  |  |  |  |
|  |  |  |  |  |
| **Supplementary table C: The biomarker C5M, mean and standard deviaton** | | | | |
|  | **Healthy donors** | **irritable bowel syndrome** | **Crohn's disease** | **Ulcerative colitis** |
| **Mean** | 5,579 | 8,136 | 8,836 | 8,604 |
| **Standard deviation** | 1,031 | 2,006 | 2,083 | 4,009 |
|  |  |  |  |  |
|  |  |  |  |  |
|  |  |  |  |  |
|  |  |  |  |  |
| **Supplementary table D: The biomarker Pro-C5, mean and standard deviaton** | | | | |
|  | **Healthy donors** | **irritable bowel syndrome** | **Crohn's disease** | **Ulcerative colitis** |
| **Mean** | 249,5 | 394,2 | 479,7 | 573 |
| **Standard deviation** | 95,02 | 143,8 | 178,4 | 197,8 |
